# Symptoms of epilepsy and organic brain dysfunctions in patients with acute, brief depression combined with other fluctuating psychiatric symptoms: a controlled study from an acute psychiatric department

**DOI:** 10.1186/1471-244X-9-63

**Published:** 2009-09-30

**Authors:** Arne E Vaaler, Gunnar Morken, Olav M Linaker, Trond Sand, Kjell A Kvistad, Geir Bråthen

**Affiliations:** 1 Department of Neuroscience, Norwegian University of Science and Technology (NTNU), Trondheim, Norway; 2 Department of Circulation and Diagnostic Imaging, NTNU, Trondheim, Norway; 3 Division of Psychiatry, Haukeland University Hospital, Bergen, Norway; 4 Division of Psychiatry, Department Østmarka, St. Olavs University Hospital, Trondheim, Norway; 5 Division of Psychiatry, Department of Research and Development, St. Olavs University Hospital, Trondheim, Norway; 6 Department of neurology and clinical neurophysiology, St. Olavs University Hospital, Trondheim, Norway; 7 Department of Diagnostic Imaging, St Olavs University Hospital, Trondheim, Norway

## Abstract

**Background:**

In psychiatric acute departments some patients present with brief depressive periods accompanied with fluctuating arrays of other psychiatric symptoms like psychosis, panic or mania. For the purpose of the present study we call this condition Acute Unstable Depressive Syndrome (AUDS).

The aims of the present study were to compare clinical signs of organic brain dysfunctions and epilepsy in patients with AUDS and Major Depressive Episode (MDE).

**Methods:**

Out of 1038 consecutive patients admitted to a psychiatric acute ward, 16 patients with AUDS and 16 age- and gender-matched MDE patients were included in the study. Using standardized instruments and methods we recorded clinical data, EEG and MRI.

**Results:**

A history of epileptic seizures and pathologic EEG activity was more common in the AUDS group than in the MDE group (seizures, n = 6 vs. 0, p = 0.018; pathologic EEG activity, n = 8 vs. 1, p = 0.015). Five patients in the AUDS group were diagnosed as having epilepsy, whereas none of those with MDE had epilepsy (p = 0.043). There were no differences between the groups regarding pathological findings in neurological bedside examination and cerebral MRI investigation.

**Conclusion:**

Compared to patients admitted with mood symptoms fulfilling DSM 4 criteria of a major depressive disorder, short-lasting atypical depressive symptoms seem to be associated with a high frequency of epileptic and pathologic EEG activity in patients admitted to psychiatric acute departments.

**Trial registration:**

NCT00201474

## Background

In psychiatric acute and intensive care units a limited number of patients presents with brief depressive periods accompanied by rapidly changing psychiatric symptoms. These patients fail to meet current diagnostic criteria for affective disorders and the optimal treatment is not established. For the purpose of the present study we call this condition Acute Unstable Depressive Syndrome (AUDS). The specific criteria are described in the methods section.

The relationships between organic brain dysfunctions and psychiatric disorders are complex [1,2]. Patients with epilepsy suffer more frequently from psychiatric illnesses than expected [3,4]. The most frequent symptoms are those indicating affective disorders and depression [5]. Affective syndromes in the epileptic population may have clinical presentations similar to affective syndromes in the non-epileptic population [4]. However, affective syndromes described in epileptic ictal, postictal or interictal periods often tend to have an atypical presentation with clinical features failing to meet current DSM-4 criteria [6] for affective disorders [5,7]. The major divergences in presentation are believed to be a brief duration of affective symptoms, and an intermixture of the depressive or manic periods with brief episodes of other psychiatric symptoms like explosive irritability, delusions, confusion and anxiety [8,9]. In a population of patients with pharmacoresistant partial epilepsy, Kanner et al. found that in most patients postictal psychiatric symptoms presented as a "clustering of various symptom categories" [10]. Recurrent brief depressive episodes have been described as core symptoms both in the presence of postictal dysphoria and interictal, dysphoric disorder in epilepsy [11,12].

Studies of epilepsy or other organic brain dysfunctions in psychiatric acute departments are sparse [12]. In an acute psychiatric care hospital, Boutros et al. found that 10% of consecutively referred inpatients had an epilepsy diagnosis [13]. Frequently, the history or presence of epilepsy was not documented in the records, and there is reason to assume that a history of convulsive disorders is often under-reported in psychiatric departments [13]. Some patients with epilepsy or other organic brain dysfunctions who are admitted to psychiatric acute wards are described as displaying fluctuating arrays of symptoms failing to fit into current nosological systems [14,15].

In some groups of patients with affective syndromes, treatment with traditional antidepressants in the absence of an antiepileptic mood-stabiliser may induce cycle acceleration [16,17]. Thus, recognition of organic brain syndromes like epilepsy in acute wards is important and may have short- and long-term therapeutic consequences [18].

The main objective of the present study was to investigate clinical signs of organic brain dysfunctions and epilepsy in acutely admitted AUDS patients compared to sex- and age-matched patients suffering from a Major Depressive Episode (MDE).

## Methods

The psychiatric department at St. Olavs University Hospital has a catchment area of 140.000 inhabitants. About 700 patients above 18 years with acute psychiatric conditions are admitted each year. Norwegian acute psychiatric services are public and available to everyone. All the patients in the catchment area are admitted to this department. Acute admissions to other psychiatric hospitals occur only if inhabitants temporarily reside outside the catchment area when the need for acute admittance arises. All patients acutely admitted during three years were evaluated for inclusion. The evaluations were performed on the first weekday after admission by experienced psychiatrists (GM or AEV).

We used the following criteria for inclusion in the AUDS group: a history of a rapidly developing psychiatric condition starting within the last 14 days. Within these 14 days the patient had at different times shown symptoms that met criteria for at least two Axis 1 diagnoses in the Diagnostic and Statistical Manual of Mental Disorders, fourth edition (DSM-4) (with exception of the time criterion) [6]. One of these diagnoses should be a depressive episode (with exception of the time criterion) defined as a score on the Montgomery and Aasberg Depression Rating Scale (MADRS) ≥20 [19]. At least one of the following criteria lead to exclusion: patients who had a psychiatric condition due to direct effects of acute intoxication, had dementia or mental disabilities to such a degree that informed consent could not be obtained, had received a diagnosis of unstable personality disorder with similar symptoms at former admissions, or were unable to speak English or Norwegian.

The control group consisted of acutely admitted sex- and age-matched patients (+/- 5 years) meeting criteria (MADRS ≥20) for a current Axis 1 major depressive episode (MDE) including the duration criterion of two weeks [6]. After inclusion of a study group patient, the first admitted patient meeting these criteria was recruited to the control group.

Written consent was obtained from all the patients prior to inclusion. The study has been performed in accordance with the ethical standards laid down in the 1964 Declaration of Helsinki. The Regional Ethical Committee approved the study.

### Assessments

Depressive symptoms were assessed with the observer rated version of the MADRS on the first weekday after admission. The MADRS has a scoring range 0-60 with scorings > 20 indicating moderate and > 30 severe depression [20]. Alcohol and illicit drug use were assessed with the Alcohol Use Disorder Identification Test (AUDIT) [21], and the Structural Clinical Interview for DSM-IV (SCID-1) [22]. The AUDIT questionnaire consists of ten items designed to identify patients with harmful alcohol use [23]. It has a scoring range 0-40 with scorings ≥8 indicating problem drinking. The questionnaire was administered as interview. The AUDIT and SCID-1 were applied as soon as the patients were able to co-operate. Urine and blood samples taken at admittance were analyzed for current drug use and medications.

The patients had three 30 minutes eyes closed EEG-videometry recordings at day 2, day 4-5 and day 8-10 after admittance. The details of the methods for the EEG recordings and the quantitative EEG (QEEG) results are published previously [24]. All patients had an MRI-scan with examinations performed at 1.5 T or 3 T magnetic field strength. The image protocols consisted of at least sagittal T1-weighted images, axial T2-weighted images, coronal FLAIR images and axial diffusion weighted images. In some cases an additional axial FLAIR-sequence was added.

Finally, after discharge from the psychiatric acute ward, the patients were referred to an experienced consultant in Neurology (GB) who had access to both EEG and MRI results from the present admission as well as all other EEGs and MRIs in the medical records. The neurologist was blinded for the grouping of the patients. He assessed various signs of organic brain pathology from the clinical examination, EEG or MRI examinations. Pathological findings at clinical examination were divided into significant (related to CNS pathology) and insignificant (e.g. a weak reflex due to previous sciatica). In EEG only regional or generalized slow activity or epileptiform activity was considered pathological.

### Statistical methods

Categorical group differences were tested with Fisher's exact tests. EEG findings were categorised as normal, focal slow, generalized slow, or epileptiform activity, allowing for more than one pathological feature in each patient. The number of patients with two or more pathological features was compared using Fisher's exact test.

## Results

In the study period 1038 patients with a total of 1984 admittances were evaluated for inclusion. Twenty-eight (2.7%) fulfilled criteria for inclusion in the AUDS group. Twelve were not included (due to non-consent, inability to consent, or language problems). Sixteen patients entered the study. The co-diagnoses in the AUDS group (with exception of the time criteria) in this group were DSM-IV 298.8 "brief psychotic disorder" (nine patients), DSM-IV 300.1 "panic disorder" (four patients) and DSM-IV 296.0 "single manic episode" (three patients). Both groups consisted of six men and ten women with mean ages 32.1 (SD 11.4) (AUDS) and 32.8 (SD 13.0) (MDE) (p = 0.99). MADRS at entrance was 29.2 (SD 8.9) in the AUDS group and 34.6 (SD 7.1) in the MDE group (p = 0.07).

Clinical background data at admittance to hospital prior to inclusion are summarised in Table 1. Seven patients from the AUDS group and none in the MDE group used anti-epileptic drugs at admittance; four for epilepsy and three for other indications. Three patients used lamotrigine, one used valproate, while three used carbamazepine. Six patients in the AUDS group and three patients in the MDE group used benzodiazepines. The indications were mainly anxiety and agitation. One patient from the MDE group had used cannabis prior to admission. There were no significant differences between the groups regarding problem drinking as defined by AUDIT scores above 8. Three of the MDE and none of the AUDS patients had withdrawal symptoms judged by clinical observations at the time of admission and through the first EEG recording.

**Table 1:** Clinical background data at admission to hospital prior to inclusion.

	**AUDS group**	**MDE group**	**p-value**
	**n = 16**	**n = 16**	

Gender	6 (38%)	6 (38%)	1.00^e^

Mean age (SD)	32.1 (11.4)	32.8 (13.0)	0.99^d^

			

Anti-epileptic medication for epilepsy	4	0	0.10^e^

Anti-epileptic medication other indications	3	0	0.23^e^

Benzodiazepine medication	6	3	0.43^e^

Problem drinking^a^	4 ^b^	4 ^c^	1,00^e^

^a ^The Alcohol Use Disorders Identification Test (AUDIT) ≥ 8

^b ^n = 15 ^c ^n = 13 ^d ^Mann-Whitney test ^e ^Fisher exact test

AUDS: Acute Unstable Depressive Syndrome

MDE: Major Depressive Episode

The clinical differences between the groups as judged by the neurologist are summarised in Table 2. Eight patients refused having a clinical neurological examination. There were significant group differences in seizures in clinical history and EEG pathology, and a significant difference with regard to number of patients fulfilling clinical criteria for epilepsy.

**Table 2:** Clinical differences between Acute Unstable Depressive Syndrome (AUDS) (n = 16) and Major Depressive Episode (n = 16) groups.

	**Valid no**.	**AUDS** **patients**	**MDE** **patients**	P value*
Seizures in clinical history	32	6/16	0/16	0.018

Fulfilling clinical criteria for epilepsy	32	5/16	0/16	0.043

Focal or generalized slow, or epileptiform EEG activity**	32	8/16	1/16	0.015

Cerebral MRI pathology (any)	26	2/13	2/13	1.0

Pathological findings at clinical neurological examination indicative of CNS pathology'	24	3/11	1/13	0.60

*Fisher exact tests.

**No of patients with ≥2 pathological features

Out of the five patients fulfilling clinical criteria for epilepsy, two had juvenile generalised epilepsy syndromes, both with generalized tonic-clonic (GTC) seizures and absences. One had a syndrome of posttraumatic localisation-related epilepsy with nocturnal seizures presumed to be secondarily generalized, and two had scattered GTCs with a syndromic classification that remained undetermined.

Two patients in each study group had pathological findings on brain MRI. In the AUDS group, one had posttraumatic loss of substance from the right temporal and frontal lobes, and the other had postoperative changes from removal of a cerebellar astrocytoma many years earlier. In the control group, one had an increased signal in pons that was interpreted as gliosis of ischemic origin. The final patient had a megacisterna magna and a somewhat uncommon appearance of vermis cerebelli.

At clinical neurological examination, three AUDS patients and one control patient had signs of CNS pathology. One AUDS patient had ophtalmoplegia and bilaterally inverted plantar reflexes (positive Babinski's sign), whereas another had inverted plantar reflexes as the only sign of pathology. The third patient had bilateral horizontal nystagmus. In the control group, there was a unilaterally inverted plantar reflex in one patient.

Most of the depressive periods in the AUDS group lasted for a few hours up to a couple of days. Five patients (31%) had very brief periods with serious affective symptoms, including intense suicidal ideations, lasting less than one hour. Their affective symptoms were agitation with panic attacks, aggression towards others and suicide attempts.

## Discussion

In the present study we compared two different groups of patients that were acutely admitted to a psychiatric acute ward with depressive symptoms. Our results indicate that patients presenting with brief depressive periods and coexisting fluctuating arrays of other psychiatric symptoms like psychosis, panic or mania, have epileptic activity more often than patients with pure major depressive episodes.

The typical contemporary psychiatric acute department patient often presents in severe crisis, complicated by substance abuse, polypharmacy, behavioural dyscontrol and multiple Axis 1 diagnoses [25]. The AUDS group patients differ from these typical patients by presenting rapidly changing different clusters of symptoms and having a short duration of the depressive episodes. A psychiatric clinical evaluation at admittance based on these clinical aspects could enable the clinician to detect patients with increased possibility of having epileptic activity. This is important information when deciding which drugs to use to rapidly tranquilize the patient. An antiepileptic mood stabilizer or a benzodiazepine would be safe with respect to the possibility of lowering the seizure threshold.

Many studies regarding epilepsy and psychiatric symptoms are population-based or stem from tertiary epilepsy centres. The study samples have consisted of patients with definite diagnoses of epilepsy in stable phases of their psychiatric conditions. Despite the high prevalence of depression in epilepsy, the treatment remains "unexplored territory" with a complete lack of double-blinded, placebo controlled studies [26]. The clinicians must rely on data available from studies of patients with primary mood disorders [9]. In the treatment of depressive symptoms, one important clinical decision is whether to use antidepressants or antiepileptic mood-stabilizers [18]. In a patient population from a tertiary epilepsy centre, Blumer argues that conditions with similar symptoms as the AUDS group require both antidepressant and antiepileptic medication [12]. The majority of antidepressants do not reduce the seizure threshold, and there is growing evidence that many antidepressants have anticonvulsant effects [27]. In the AUDS group the volatility of symptoms and the obvious need for affective stabilization and control of behaviour are factors favouring the initial use of fast acting antiepileptic mood-stabilizers, although empirical confirmation of this assumption is needed. If prophylactic long-term treatment is indicated, an antiepileptic mood-stabiliser with effects both on the affective disorder and the seizures is also a reasonable choice [28]. However, empirical confirmation is again lacking. Empirically based information about the best choices is much needed due to the probably increasing number of patients suffering from organic brain disorders who are admitted involuntarily to acute and emergency services [29].

This study has a number of limitations. The patients were recruited in daily, routine clinical practice at admittance to the acute ward. It is difficult to imagine how a blinded research design regarding inclusion could be applied in such a setting. When in doubt whether criteria for inclusion were fulfilled or not, both senior psychiatrists (GM and AEV) did individual evaluations of the patients. Patients were recruited when both psychiatrists found the criteria to be fulfilled. The neurologist (GB), radiologist (KAK) and neurophysiologist (TS) were all blinded for patients' group allocations.

The groups were composed of patients with substance abuse, withdrawal symptoms, and medication with antiepileptic drugs or benzodiazepines. These factors affect the expression of symptoms as well as EEG interpretations. To obtain a more naturalistic study, we chose not to exclude patients with substance use diagnoses due to the substantial number of patients in acute departments with these conditions [30]. More patients in the AUDS group than in the MDE group used antiepileptic drugs or benzodiazepines at admittance. These medications may have decreased the pathological findings in EEG recordings and stabilised clinical symptoms. Thus, potential EEG pathology may have been masked to a greater extent in the AUDS group than in the MDE group.

There are several strengths of the study. First of all this is a prospective study of a naturalistic patient population from a defined catchment area. All patients admitted in a three year period were evaluated for inclusion. We used robust validated instruments assessing diagnoses, symptoms of depression and alcohol abuse. All patients had urine and blood screens, assessing substance abuse and medication, and EEG was performed shortly after admittance.

The small number of subjects leading to relatively weak statistical power could be held against the study. The results that several indicators of brain pathology still reached statistical significance may indicate a fairly strong association between organic brain syndromes, epilepsy and the AUDS clinical picture.

We have called our study group AUDS, pinpointing the acute and unstable, depressive core symptoms. Patients displaying similar symptoms have been described in the literature with different names like "masked epilepsy", "temporal lobe syndrome", "interictal dysphoric disorder", and "subictal dysphoric disorder" [12]. These names are applied to describe conditions characterised by seizures or dysfunctions presumably originating in or primarily involving mesial temporal limbic structures. Thus, a close collaboration between neurologists and psychiatrists in the evaluation and management of these patients is appropriate. However, such collaboration is rarely encountered, even in tertiary epilepsy centres [31], and even less in psychiatric acute wards and psychiatric intensive care units. Adequate treatment and evaluation of people with psychiatric conditions require that neurological conditions are recognised and incorporated into the overall treatment.

## Conclusion

Patients in psychiatric acute departments presenting symptoms characterised by acute, brief depression combined with other fluctuating psychiatric symptoms, have more seizures in clinical history, and more focal, generalized slow, or epileptiform activity compared to sex and age matched patients acutely admitted with Major Depressive Episodes.

## Competing interests

Dr Morken has received a travel grant from Astra Zeneca, but none of the authors have any financial interests or other potential conflicts of interest.

The study received funding from Glaxo SmithKline (GSK).

## Authors' contributions

AEV, GM, OML and GB conceived, designed, and coordinated the study, examined and included the patients and helped to draft the manuscript. TS planned and supervised the EEG procedures and interpreted the EEGs. KAK planned and supervised the MRI procedures and interpreted the MRIs. All authors read and approved the final manuscript.

## Pre-publication history

The pre-publication history for this paper can be accessed here:


